# IFN-*γ*-Licensed Mesenchymal Stem Cells Are More Susceptible to Death when Exposed to Quorum-Sensing Signal Molecule OdDHL and Less Effective in Inhibiting the Growth of *Pseudomonas aeruginosa*

**DOI:** 10.1155/2024/2934308

**Published:** 2024-07-30

**Authors:** Marielly Reis Resende Sousa, Amandda Évelin Silva-Carvalho, Maurício Gonçalves da Costa Sousa, Danilo César Mota Martins, Emãnuella Melgaço Garcez, Luma Dayane de Carvalho Filiú Braga, Juliana Lott de Carvalho, Tanise Vendruscolo Dalmolin, Taia Maria Berto Rezende, Felipe Saldanha-Araujo

**Affiliations:** ^1^ Laboratory of Hematology and Stem Cells (LHCT) Health Sciences Department University of Brasilia, Brasília, DF, Brazil; ^2^ Molecular Pharmacology Laboratory Health Sciences Department University of Brasília, Brasilia, DF, Brazil; ^3^ Genomic Sciences and Biotechnology Program Catholic University of Brasília, Brasilia, DF, Brazil; ^4^ Division of Biomaterials and Biomechanics Department of Restorative Dentistry School of Dentistry Oregon Health and Science University, Portland, OR, USA; ^5^ Knigth Cancer Precision Biofabrication Hub Knigth Cancer Institute Oregon Health and Science University, Portland, OR, USA; ^6^ Cancer Early Detection Advanced Research Center Oregon Health Science University, Portland, OR, USA; ^7^ Health Sciences Postgraduate Program University of Brasília, Brasília, DF, Brazil; ^8^ Dentistry Course Catholic University of Brasília, Brasília, DF, Brazil; ^9^ Multidisciplinary Laboratory of Biosciences Faculty of Medicine University of Brasilia, Brasilia, DF, Brazil; ^10^ Clinical Analysis Laboratory Faculty of Health Sciences University of Brasília, Brasília, DF, Brazil; ^11^ Department of Dentistry Faculty of Health Sciences University of Brasília, Brasília, DF, Brazil

## Abstract

Currently, a series of licensing strategies has been investigated to enhance the functional properties of mesenchymal stem cells (MSCs). Licensing with IFN-*γ* is one of the most investigated strategies for enhancing the immunosuppressive potential of such cells. However, it is not yet known whether this licensing strategy could interfere with the ability of MSCs to control bacterial growth, which may be relevant considering their clinical potential. In this study, we compared the antimicrobial potential of IFN-*γ*-licensed and unlicensed MSCs by exposing them to *Pseudomonas aeruginosa* and its quorum-sensing inducer molecule OdDHL. Our data show that—when challenged with OdDHL—IFN-*γ*-licensed and unlicensed MSCs present increased levels of the antimicrobial *HAMP* transcript, but that only IFN-*γ*-licensed MSCs undergo modulation of *CASP1* and *BCL2*, entering apoptosis. Furthermore, we demonstrate that only IFN-*γ*-licensed MSCs show modulation in genes involved in apoptosis and tend to undergo cell death when cultured with *P. aeruginosa*. As a consequence, IFN-*γ*-licensed MSCs showed lower capacity to control bacterial growth, compared to unlicensed MSCs. Taken together, our observations reveal an increased susceptibility to apoptosis of IFN-*γ*-licensed MSCs, which compromises their potential to control the bacterial growth *in vitro*. These findings are relevant to the field of cell therapy, considering the potential applicability of MSCs.

## 1. Introduction

More recently, several studies have been conducted to seek licensing approaches (*priming*) that can enhance the biological properties of mesenchymal stem cells (MSCs) [1]. Among the different strategies investigated, the licensing of MSCs with IFN-*γ* is the most explored. Interestingly, IFN-*γ*-licensed MSCs show greater expression of adhesion molecules with immunomodulatory potential, generate more regulatory lymphocytes, and show greater production of soluble molecules with immunosuppressive potential. Furthermore, it has been shown that IFN-*γ* licensing decreases cryopreserved MSC's susceptibility to lysis by T cells [2].

As licensed MSCs approach clinical use, it becomes increasingly relevant to explore the impact of MSC priming on other MSC properties. For instance, in clinical settings, it is possible that such cells are infused in patients with opportunistic infections and even sepsis [3, 4]. Although MSCs have antimicrobial properties, their interaction with bacteria is complex. For example, TLR4 signaling by bacterial products on MSC surface can change their phenotype to a proinflammatory profile [5]. Therefore, the interaction between unlicensed and licensed MSCs with the microbial microenvironment needs to be further investigated.

*Pseudomonas aeruginosa* is an important nosocomial pathogen, known for its ability to be resistant to antibiotic treatments and is associated with a higher incidence of multidrug-resistant infections in hospital settings and an increased risk of mortality [6, 7]. In particular, immunocompromised patients, such as individuals with grade 3–4 graft-versus-host disease, have a higher risk of recurrent infection [8]. Importantly, MSCs are used in the treatment of GVHD mainly due to their immunomodulatory properties [9, 10]. Nevertheless, given that MSCs have been shown to exhibit antimicrobial activity against *P. aeruginosa in vitro* and *in vivo* [11, 12], their therapeutic effects might also involve antimicrobial response. It is important to note that the immunomodulatory properties of MSCs could be enhanced by licensing with IFN-*γ*; however, it is still unknown whether this licensing strategy could modulate the antimicrobial potential of these cells.

Another point that deserves attention in this context is that MSCs seem to be sensitive to quorum-sensing molecules produced by bacteria [13]. Interestingly, bacteria use quorum-sensing communication circuits to regulate physiological activities such as symbiosis, virulence, motility, and biofilm formation [14]. A previous report demonstrated that the *P. aeruginosa*-derived quorum-sensing signaling molecules OdDHL (N-3- (oxododecanoyl)-l-homoserine lactone) and HHQ (2-heptyl-4-quinolone) can modulate cytokine production and induce MSC death [13]. However, there is a lack of information in the literature regarding the effect of quorum-sensing signaling molecules on IFN-*γ*-licensed MSCs.

Considering the applicability of IFN-*γ*-licensed MSCs and the lack of knowledge regarding their antimicrobial properties, in this work, we investigated whether IFN-*γ* licensing could confer a protective effect on MSCs exposed to the quorum-sensing signal molecule OdDHL and whether this licensing strategy could modulate the ability of MSCs to control *P. aeruginosa* growth.

## 2. Material and Methods

### 2.1. MSC Culture, Characterization, and Licensing

MSCs were obtained from healthy donors (*n* = 3) following a lipoaspiration procedure [15]. The cells were cultured in alpha-minimum essential medium (*α*-MEM) supplemented with 15% fetal bovine serum (FBS) (HyClone, Logan, UT, USA), 2 mM glutamine, and 100 U/mL penicillin/streptomycin (Sigma–Aldrich, St. Louis, MO, USA), at 37°C and 5% CO_2_. The medium was changed every 2 days, and the cells were split when they reached 80%–90% confluence.

MSCs were phenotypically characterized by flow cytometry (FACSCalibur, BD Biosciences, Franklin Lakes, NJ, USA) using the BD Stemflow™ hMSC Analysis Kit, following the manufacturer's instructions (Pharmingen, BD Biosciences, Franklin Lakes, NJ, USA). Ten thousand events were recorded for each sample, and data were analyzed using FlowJo software 10.0.7 (Treestar Inc.). This kit includes anti-CD105, anti-CD73, anti-CD90, and anti-CD44 antibodies and has a negative cocktail with anti-CD45/CD34/CD11b/CD19/HLA-DR. Adipogenic, osteogenic, and chondrogenic MSC differentiation potential was evaluated, as previously described [16, 17].

MSC licensing was performed following their incubation for 48 hr with 50 ng·mL^−1^ of IFN-*γ* [18]. After treatment, cells were washed with PBS three times before the beginning of the experiments. MSCs from the fourth to sixth passage were used for experiments. The study was approved by the Ethical Committee of Health Sciences Faculty of the University of Brasília (64079216.3.3001.0026).

### 2.2. N-(3-Oxododecanoyl)-L-Homoserine Lactone (OdDHL)

Quorum-sensing signal molecule OdDHL was purchased from (Sigma–Aldrich), solubilized in DMSO, and stored at −20°C. For the proposed experiments, control (untreated) and IFN-*γ*-licensed MSCs were exposed to the OdDHL at concentrations ranging from 0.5 to 50 *μ*M, as described below.

### 2.3. MTT Assay

We investigated the effect of OdDHL on the viability of unlicensed MSC and IFN-*γ*-licensed MSCs using the MTT (3-(4.5-dimethylthiazol-2-yl)-2,5-diphenyl tetrazolium bromide) assay, as previously described [19]. For this, MSCs were incubated for 24 hr with 0.5, 1, 10, or 50 *μ*M OdDHL. After this period, cells were incubated with 0.5 mg/mL MTT for 3 hr. Then, MTT and medium were removed and replaced by DMSO. The plate was homogenized for 15 min, and the optical density was read on a Multiskan FC Plate Reader (Thermo Fisher, Massachusetts, USA) at 570 nm.

### 2.4. Apoptosis Assay

The apoptotic effect of OdDHL on MSCs was determined by annexin V/propidium iodide (PI) staining, using flow cytometry. For this, unlicensed MSC and IFN-*γ*-licensed MSCs were incubated for 24 hr with OdDHL (10 or 50 *μ*M) or *P. aeruginosa*. Then, cells were trypsinized and stained with annexin V-FITC and PI, according to the manufacturer's instructions. The analyses were performed using the FlowJo software 10.0.7 (FlowJo LLC, USA). Ten thousand events were recorded for each sample. Viable (annexin V^−^/PI^−^) early apoptotic (annexin V^+^/PI^−^) and late apoptotic cells (annexin V^+^/PI^+^) were quantified using FlowJo software 10.0.7 (FlowJo LLC, USA).

### 2.5. Measurement of Mitochondrial Membrane Potential

The effect of OdDHL on the mitochondrial membrane potential of MSCs was determined by incubating these cells with the lipophilic cationic dye rhodamine 123 (5 *μ*g·mL^−1^). Unlicensed MSC and IFN-*γ*-licensed MSCs were incubated for 24 hr with 50 *μ*M OdDHL in a 6-well plate. After this period, cells were recovered and stained with rhodamine 123 for 20 min, and the fluorescence was detected by flow cytometry (FACSCalibur, BD Biosciences, Franklin Lakes, NJ, USA). Ten thousand events were recorded for each sample.

### 2.6. Lactate Dehydrogenase (LDH) Release Assay

LDH release was determined using the kit CytoTox 96 Non-Radioactive Cytotoxicity Assay, following the manufacturer's instructions (Promega Corp., Madison, WI, USA). Briefly, unlicensed MSC and IFN-*γ*-licensed MSCs were incubated for 24 hr with 50 *μ*M OdDHL in a 96-well plate. After this period, the supernatant was transferred to another 96-well plate, and the CytoTox 96 solution was added to each well. The plate was incubated for 30 min, and then, 50 *μ*L of the stop solution was added to each well. The plate was homogenized, and the optical density was read on a Multiskan FC Plate Reader (Thermo Fisher, Massachusetts, USA) at 490 nm.

### 2.7. Caspase 3/7 Activity

Caspase 3/7 activity was determined using the kit Caspase-Glo 3/7 Assay, following the manufacturer's instructions (Promega Corp., Madison, WI, USA). Briefly, unlicensed MSC and IFN-*γ*-licensed MSCs were incubated for 24 hr with 50 *μ*M OdDHL in a white 96-well plate. After this period, 100 *μ*L of Caspase-Glo 3/7 reagent was added per well, and the plate was kept at room temperature for 2 hr. Then, the luminescence generated was determined on a Multimode Plate Reader (PerkinElmer, Waltham, MA, USA).

### 2.8. Peripheral Blood Mononuclear Cell (PBMC) Isolation and Immunosuppression Assay

The immunosuppression experiments were conducted in an allogeneic context, with different MSC and PBMC donors. PBMCs were obtained from two healthy volunteers by centrifugation using histopaque 1,077 (Sigma–Aldrich). After isolation, PBMCs were activated with 5 *μ*g·mL^−1^ of phytohaemagglutinin (PHA, Sigma–Aldrich) and used in coculture experiments after being stained with 2.5 *μ*M carboxyfluorescein succinimidyl ester (CFSE).

To evaluate the effects of OdDHL on the immunosuppressive effect of unlicensed MSC and IFN-*γ*-licensed MSCs, these cells were cocultured with PBMCs for 3 days, and 0.5 or 1 *μ*M OdDHL was added on the first day of the experiment. As a control, the effect of OdDHL on the proliferation of PHA-activated T cells cultured alone was also determined. On the third day, PBMCs were collected and incubated with anti-CD3-APC (Thermo Fisher), and the proliferation of CFSE-labeled T cells was determined by flow cytometry.

### 2.9. Real-Time PCR

Gene expression analysis was performed in unlicensed MSC and IFN-*γ*-licensed MSCs after their exposure to OdDHL (1 and 50 *μ*M) or *P. aeruginosa*. RNA samples were obtained using TRIzol reagent (Thermo Fisher). RNA amount and quality were determined by the NanoDrop One Spectrophotometer (Thermo Fisher). One microgram of RNA was converted to single-stranded cDNA, using the High-Capacity Kit (Applied BioSystems, Foster City, CA, USA), according to the manufacturer's recommendations. mRNA expression levels of the selected genes were determined by real-time PCR (Applied Biosystems StepOnePlus™) with SYBR Green Master Mix (Thermo Fisher, USA) combined with primers specific to each gene (*supplementary table 1*). GAPDH was used as internal reference. Amplification reactions were performed in duplicates, and the relative fold value was obtained by the 2^−*ΔΔ*Ct^ method [20].

### 2.10. Antimicrobial Assay with *P. aeruginosa*

Cultures of *P. aeruginosa* (ATCC 27853) were prepared in Luria-Bertani medium (Difco, BD Biosciences) at 37°C with slight agitation. Before the experiments, the bacteria were washed, resuspended in PBS, and the optical density (OD at *λ* = 600 nm) of the suspension was measured. The number of colony-forming units (CFU) was calculated according to the following equation: OD 600 = 0.3 is equivalent to 5 × 10^11^ CFUs·mL^−1^.

To assess the antimicrobial potential of unlicensed MSC and IFN-*γ*-licensed MSCs, 2.6 ×10^4^ cells/cm^2^ were seeded in a 6-well plate, and immediately after the licensing with IFN-*γ* (48 hr), a cell suspension of 5.10^4^ CFUs·mL^−1^*P. aeruginosa* in *α*-MEM medium supplemented with 5% FBS (no antibiotics) was added to each well. The plate was incubated in a humidified CO_2_ incubator at 37°C for 10 hr. After this period, samples were diluted (1 : 10,000), and CFUs were manually counted.

### 2.11. Statistical Analysis

Data were reported as mean ± SEM, and at least three independent experiments were performed. All analyses were performed using Prism 9 software (GraphPad Software Inc., San Diego, CA, USA). Differences between two groups were analyzed by a nonparametric Mann–Whitney test. ANOVA, Kruskal–Wallis test, and Dunn's multiple comparisons were used for comparisons including three or more groups. Differences were considered statistically significant at *p*  < 0.05.

## 3. Results

### 3.1. MSC Characterization

**Figure 1:**
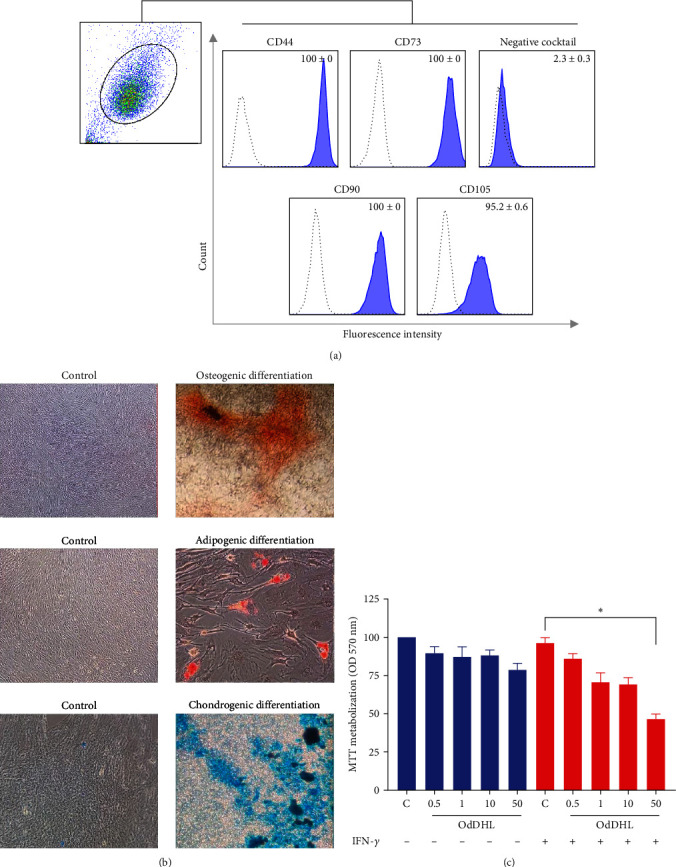
MSC characterization and the effect of OdDHL on their viability. (a) Representative flow cytometry histograms showing the immunophenotypic characterization of MSCs. (b) Differentiation of MSCs into osteocyte, chondrocyte, and adipocyte lineages. (c) IFN-*γ*-licensed MSCs tretated with 50 *µ*M of OdDHL presented decreased viability detected by MTT assay.  ^*∗*^*p* < 0.05.

MSCs presented a typical MSC immunophenotype, with positive expression of CD44 (100%), CD73 (100%), CD90 (100%), and CD105 (95.2%), and lack of CD11b, CD19, CD34, CD45, and HLA-DR markers (Figure 1(a)). Furthermore, MSCs showed capacity for differentiation into adipocytes, chondrocytes, and osteocytes (Figure 1(b)).

### 3.2. OdDHL Impacts the MSC's Viability

Using the MTT assay, we demonstrated that after 24 hr of cell culture, 50 *µ*M of OdDHL compromised significantly the viability of IFN-*γ*-licensed MSCs (*p*=0.01) (Figure 1(c)).

### 3.3. OdDHL Induces MSC Apoptosis Independent of INF-*γ* Licensing

**Figure 2:**
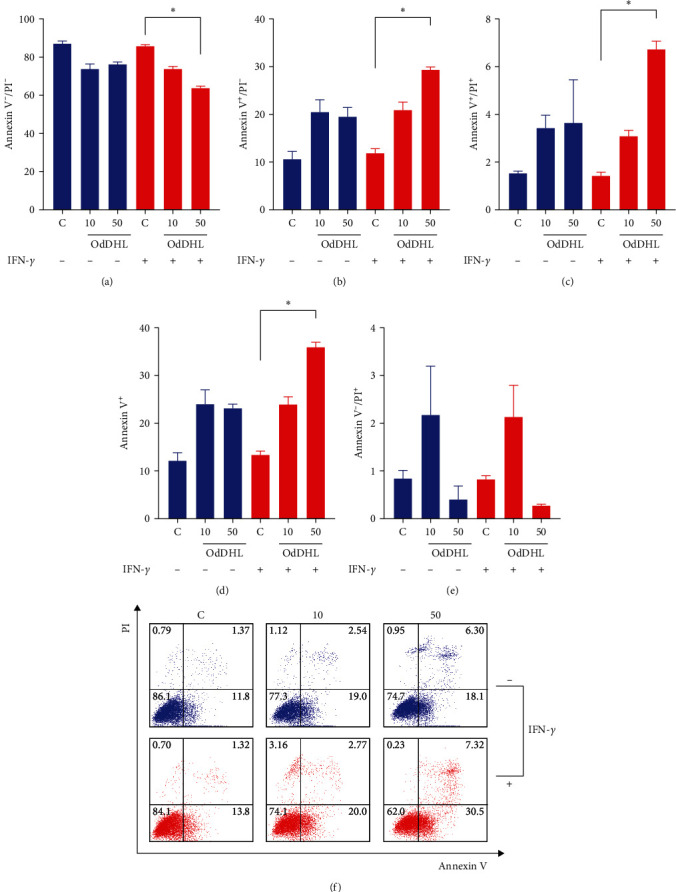
Flow cytometry assay to determine the potential of OdDHL to induce apoptosis in MSCs. Flow cytometry data show the percentage of (a) annexin V^−^/PI^−^, (b) annexin V^+^/PI^−^, (c) annexin V^+^/PI^+^, (d) annexin V^+^, and (e) annexin V^−^, PI^+^ MSCs exposed or not to OdDHL. (f) Representative dot plot of annexin V and PI expression in MSCs exposed to OdDHL. Results are presented as mean ± SEM. Asterisks indicate results that were statistically significant.  ^*∗*^ means *p* < 0.05.

We evaluated by flow cytometry whether OdDHL could induce MSC apoptosis. IFN-*γ*-licensed MSCs showed reduced cell viability when exposed to OdDHL at 50 *μ*M (*p*=0.02) (Figure 2(a)). Indeed, after exposure to 50 *μ*M OdDHL, there was a significant induction of early (*p*=0.02) and late (*p*=0.04) apoptosis in IFN-*γ*-licensed MSCs (Figures 2(b) and 2(c)). These data clearly showed that IFN-*γ*-licensed MSCs were more sensitive to the toxic effect promoted by 50 *μ*M of OdDHL (*p*=0.02) (Figure 2(d)). We found no statistically significant differences regarding the percentage of PI positive cells (Figures 2(e) and 2(f)).

### 3.4. OdDHL Does Not Alter the Mitochondrial Membrane Potential of MSCs

**Figure 3:**
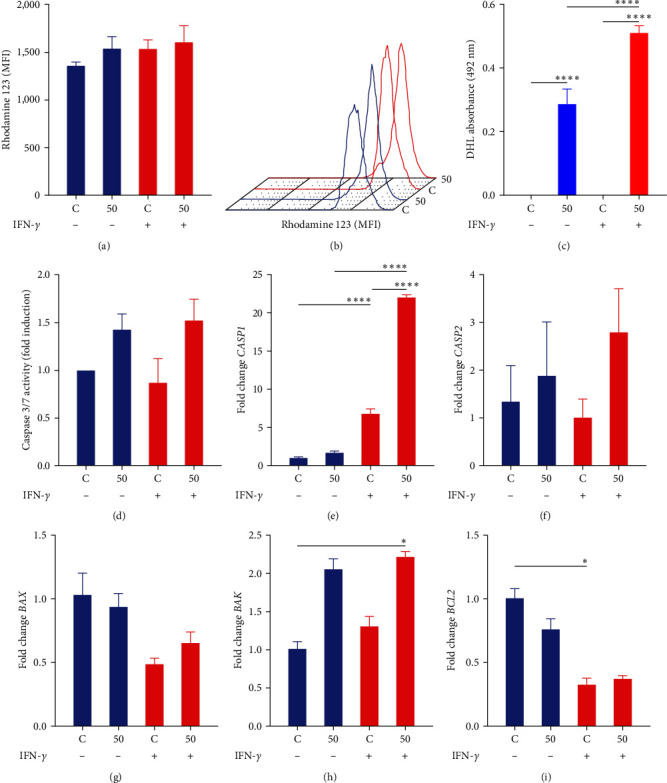
Characterization of cell death induced by OdDHL. (a) Rhodamine 123 staining in unlicensed MSC and IFN-*γ*-licensed MSCs exposed to OdDHL. (b) Representative rhodamine 123 histograms of unlicensed MSC (blue) and IFN-*γ*-licensed MSCs (red) exposed to OdDHL. (c) LDH release in culture medium of unlicensed MSC and IFN-*γ*-licensed MSCs exposed to OdDHL. (d) Caspase 3/7 activity in unlicensed MSC and IFN-*γ*-licensed MSCs exposed to OdDHL. (e–i) Transcriptional levels of *CASP1*, *CASP2*, *BAX*, *BAK*, and *BCL2* in unlicensed MSC and IFN-*γ*-licensed MSCs exposed to OdDHL. Results are presented as mean ± SEM. Asterisks indicate results that were statistically significant.  ^*∗*^ means *p* < 0.05,  ^*∗∗∗∗*^*p* < 0.0001.

We evaluated by flow cytometry whether OdDHL could promote mitochondrial dysfunction in MSCs. The exposure of MSCs to 50 *µ*M of OdDHL did not alter the mitochondrial membrane potential in these cells, regardless of IFN-*γ* licensing (Figures 3(a) and 3(b)).

### 3.5. LDH Release in MSC Exposed to OdDHL

We evaluated the release of the LDH enzyme in MSCs exposed to OdDHL. High levels of LDH were observed in unlicensed MSC (*p*  < 0.0001) and IFN-*γ*-licensed MSCs (*p*  < 0.0001) when they were exposed to 50 *µ*M of OdDHL. However, exposure to 50 *µ*M of OdDHL induces greater LDH release in IFN-*γ*-licensed MSCs (*p*  < 0.0001) than in unlicensed MSC (*p*  < 0.0001) (Figure 3(c)).

### 3.6. Caspase 3/7 Activity in MSCs Exposed to OdDHL and Levels of Apoptotic-Related Transcripts

To better understand the process of cell death induced by OdDHL, we evaluated the activity of caspase 3/7 in MSCs exposed to this quorum-sensing molecule. We did not observe statistically significant changes in the activity of caspase 3/7 in unlicensed MSC treated with 50 *µ*M of OdDHL. However, it is important to note that OdDHL increased by 42.4% (average) the activity of caspase 3/7 in unlicensed MSC, and in IFN-*γ*-licensed MSCs, this increase was 75.4% in comparison to MSCs licensed and unexposed to OdDHL (Figure 3(d)).

After evaluating the activity of caspase 3/7 in MSCs, we investigated the expression of *CASP1*, *CASP2*, *BAX*, *BAK*, and *BCL-2* transcripts in these cells. Exposure of IFN-*γ*-licensed MSCs to OdDHL significantly increased transcriptional levels of *BAK* (*p*=0.02), compared to unlicensed MSC. On the other hand, we identified a large increase in the expression of the proapoptotic factor *CASP1* in IFN-*γ*-licensed MSCs, in comparison to control MSCs (*p* < 0.0001). More importantly, this increase in *CASP1* expression is enhanced when IFN-*γ*-licensed MSCs are exposed to OdDHL (*p* < 0.0001) but not when unlicensed MSC are exposed to this quorum-sensing signal molecule. Interestingly, we identified a significant reduction in the expression of the antiapoptotic factor *BCL-2* when comparing unlicensed MSC with IFN-*γ*-licensed MSCs (*p*=0.03). No statistically significant changes were observed regarding the expression of *BAX* and *CASP2* in the groups analyzed (Figures 3(e), 3(f), 3(g), 3(h), and 3(i)).

### 3.7. Effects of OdDHL on the Immunosuppressive Potential of MSCs and T-Cell Proliferation

**Figure 4:**
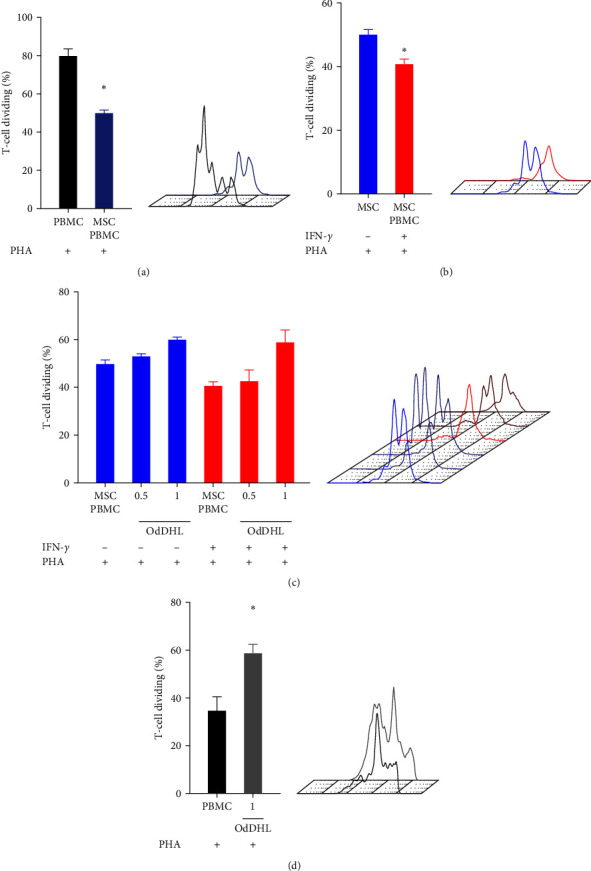
Effect of OdDHL on the immunosuppressive potential of MSCs. (a) Unlicensed MSC was cocultured with PHA-activated PBMCs (1 : 10 ratio), and T-cell proliferation was determined by flow cytometry after 3 days. (b) T-cell proliferation after the PHA-activated PBMCs coculture with unlicensed MSC or IFN-*γ*-licensed MSCs. (c) T-cell proliferation after the PHA-activated PBMC exposure to OdDHL and unlicensed MSC or IFN-*γ*-licensed MSCs. (d) T-cell proliferation after the PHA-activated PBMC exposure to OdDHL. These experiments were performed with PBMCs from two different donors. Representative histograms of an evaluated sample are presented on the right of each graph. Results are presented as mean ± SEM. Asterisks indicate results that were statistically significant.  ^*∗*^ means *p* < 0.05.

We investigated the effect of OdDHL on the immunosuppressive capacity of MSCs, considering concentrations of this quorum-sensing signal molecule that did not show cytotoxicity for these cells. As expected, MSCs were able to decrease T-cell proliferation (*p*=0.05) (Figure 4(a)), and IFN-*γ*-licensed MSCs showed a more potent immunosuppressive effect than unlicensed MSC (*p*=0.05) (Figure 4(b)). When evaluating the impact of OdDHL on the immunosuppressive effect of MSCs, we did not identify statistically significant increases in T-cell proliferation, although the proliferation of T cells co-cultured with unlicensed MSC increased on average by 20.2% when they were exposed to 1 *µ*M of OdDHL. In coculture with IFN-*γ*-licensed MSCs, T-cell proliferation increased by 44.6% in the presence of 1 *µ*M of OdDHL (Figure 4(c)). Importantly, in the absence of MSCs, OdDHL was able to significantly stimulate T-cell proliferation (*p*=0.05) (Figure 4(d)).

### 3.8. Gene Expression of MSCs Exposed to OdDHL

**Figure 5:**
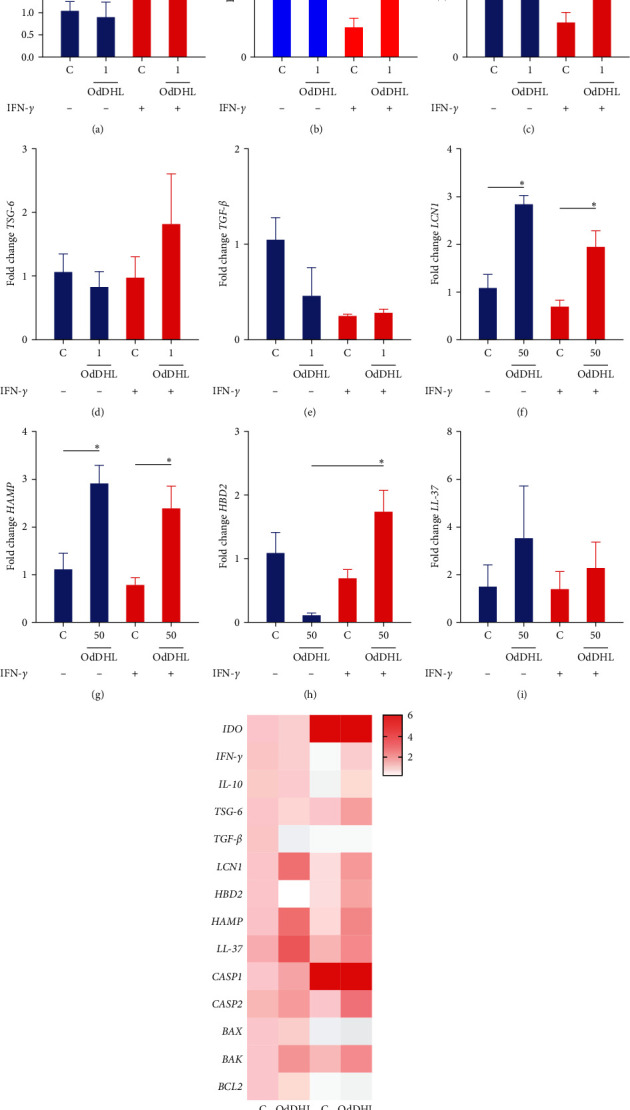
Gene expression analysis of the selected transcripts. Unlicensed MSCs and IFN-*γ*-licensed MSCs were cultured in the presence or absence of OdDHL for 24 hr and profiled by real-time PCR according to (a–e) proinflammatory and anti-inflammatory factors (*IDO*, *IFN-γ*, *IL-10*, *TSG-6*, and *TGF-β*) and to (f–i) genes encoding antimicrobial proteins (*LCN1*, *HAMP*, *HBD2*, and *LL-37*). (j) Heatmap illustrating the qRT-PCR analysis of antimicrobial-, inflammatory-, and apoptotic-related genes in unlicensed MSCs and IFN-*γ*-licensed MSCs exposed or not to OdDHL. The relative fold values were obtained by the 2^−*ΔΔ*Ct^ method, using the median Ct value of control MSCs as a reference. Results are presented as mean ± SEM. Asterisks indicate results that were statistically significant.  ^*∗*^ means *p* < 0.05,  ^*∗∗∗∗*^*p* < 0.0001.

IFN-*γ*-licensed MSCs showed a significant increase in *IDO* expression when compared to unlicensed MSC (*p*  < 0.0001) (Figure 5(a)). We did not find statistically significant differences when evaluating the expression of *IFN-γ*, *IL-10*, *TSG-6*, and *TGF-β* in unlicensed MSC and licensed MSCs with IFN-*γ*, submitted or not to OdDHL (Figures 5(b), 5(c), 5(d), and 5(e).

Interestingly, we noticed that both the unlicensed MSC and IFN-*γ*-licensed MSCs showed increased expression of *HAMP* and *LCN1* after exposure to OdDHL (Figures 5(f) and 5(g)). Furthermore, exposure to OdDHL induced increased transcriptional levels of *HBD2* in IFN-*γ*-licensed MSCs, compared to unlicensed MSCs exposed to OdDHL (*p*=0.02) (Figure 5(h)). Finally, we did not observe statistically significant transcriptional changes in *LL-37* levels (Figure 5(i)).

### 3.9. IFN-*γ*-Licensed MSCs Are More Susceptible to Death and Less Effective in Controlling the Growth of *P. aeruginosa*

**Figure 6:**
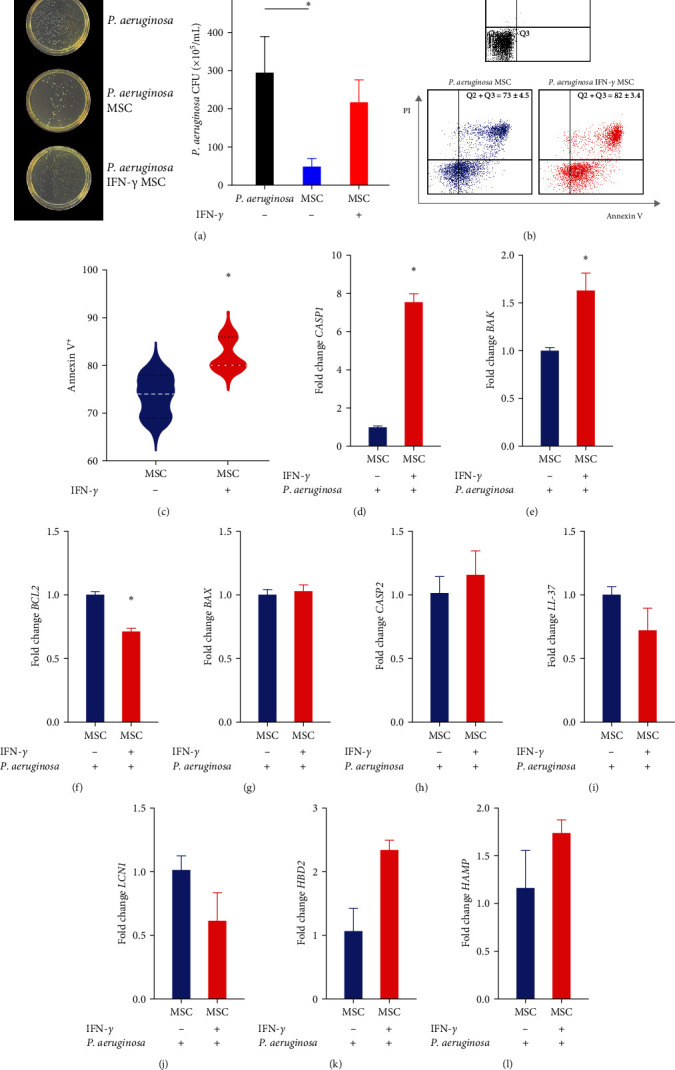
Antimicrobial potential of MSCs and the impact of *P. aeruginosa* on these cells. (a) Colony formation of *P. aeruginosa* treated with unlicensed MSCs and IFN-*γ*-licensed MSCs for 10 hr. (b) Representative dot plot with percentage (mean ± SD) of annexin V^+^ cells exposed or not to *P. aeruginosa*. (c) Annexin V^+^ unlicensed MSCs and IFN-*γ*-licensed MSCs after their culture with *P. aeruginosa*. (d–h) Gene expression analysis of apoptotic transcripts (*CASP1*, *CASP2*, *BAX*, *BAK*, *and BCL2*) and (i–l) genes encoding antimicrobial proteins (*LCN1*, *HBD2*, *HAMP*, and *LL-37*). The relative fold values were obtained by the 2^−*ΔΔ*Ct^ method, using the median Ct value of unlicensed MSCs as a reference. Results are presented as mean ± SEM. Asterisks indicate results that were statistically significant.  ^*∗*^ means *p* < 0.05.

When evaluating the antimicrobial potential of MSCs, we noticed that only unlicensed MSC was able to significantly inhibit the growth of *P. aeruginosa* (*p*=0.05) (Figure 6(a)). Interestingly, IFN-*γ*-licensed MSCs showed increased apoptosis when exposed to *P. aeruginosa* (*p*=0.02) (Figures 6(b) and 6(c)), which agrees with the increased transcriptional levels of *CASP-1* (*p*=0.05) and *BAK* (*p*=0.05) in these cells (Figures 6(d) and 6(e)). Furthermore, IFN-*γ*-licensed MSCs exposed to *P. aeruginosa* showed reduced expression of the antiapoptotic factor *BCL-2* (*p*=0.05) (Figure 6(f)). No changes in *CASP2* and *BAX* expression were identified (Figures 6(g) and 6(h)). Finally, the contact of MSCs with *P. aeruginosa* did not promote modulation in any of the genes that encode antimicrobial proteins (Figures 6(i), 6(j), 6(k), and 6(l).

## 4. Discussion

In this study, we investigated the antimicrobial potential of IFN-*γ*-licensed MSCs by exposing them both to the *P. aeruginosa* and the quorum-sensing inducer molecule OdDHL. Importantly, our data show that IFN-*γ*-licensed MSCs are more susceptible to apoptosis when exposed to OdDHL compared to their unlicensed counterparts. More importantly, we demonstrate that when IFN-*γ*-licensed MSCs are cultivated with the bacteria *P. aeruginosa*, they tend to undergo apoptosis, which compromises their potential to control the growth of such bacteria.

*P. aeruginosa* has four main quorum-sensing systems that are interconnected, the Las, Rhl, Pqs, and Iqs. OdDLH integrates the autoinducer molecules responsible for controlling the Las and Rhl systems and modulating the bacterium's virulence genes [21]. Interestingly, it has been demonstrated that the OdDHL molecule can modulate several mammalian cell functions, including immunomodulation and cell death. Holban and colleagues demonstrated that OdDHL at a concentration of 50 *µ*M was able to induce apoptosis in bone marrow MSCs, in addition to modulating the production of inflammatory factors by these cells [14]. Using the same concentration of OdDHL, we did not find a significant apoptotic effect of this molecule on unlicensed MSC. On the other hand, IFN-*γ*-licensed MSCs were more sensitive to contact with OdDHL, entering apoptosis. Accordingly, after contact with OdDHL, IFN-*γ*-licensed MSCs produced elevated levels of LDH and had on average caspase 3/7 activity increased by 75%, compared to IFN-*γ*-licensed MSCs that were not exposed to OdDHL. In addition, these cells showed high transcriptional levels of *CASP1* and *BAK* and inhibition of the antiapoptotic factor *BCL-2*. Taken together, these data show that IFN-*γ*-licensed MSCs are more susceptible to apoptosis and indicate the possibility of adipose-derived MSCs are more resistant to OdDHL-induced cell death, compared to bone marrow MSCs.

In addition to inducing IL-1*β* and IL-8 production in MSCs [14], OdDHL has been shown to exert a wide variety of immunological modulations, including stimulating neutrophil chemotaxis and the production of inflammatory factors by endothelial and epithelial cells [22]. Considering the immunomodulatory effects of MSCs, we evaluated whether, at nontoxic doses, OdDHL could modulate the ability of MSCs to control T-cell proliferation. Although the findings were not statistically significant, the presence of OdDHL in the coculture of PBMCs with IFN-*γ*-licensed MSCs or unlicensed MSCs slightly increased T-cell proliferation. However, we did not identify any modulation in genes that encode classic anti-inflammatory factors of MSCs, such as *IDO*, *TGF-β*, *IL-10*, and *TSG6* after exposing such cells to OdDHL. On the other hand, direct exposure of PBMCs to OdDHL was able to stimulate T-cell proliferation. The effect of OdDHL on the immune response seems to be dependent on its concentration [23]. When present in high doses, above 70 *µ*M, OdDHL seems to inhibit T-cell proliferation [24]. These data indicate that the immunosuppression of T cells by MSCs may be impacted depending on the amount of OdDHL present in the environment.

Cathelicidin LL-37, Lipocalin, *β*-defensin-2, and hepcidin are the main AMPs produced by MSCs [25]. In order to assess whether the OdDHL molecule exerts any regulatory impact on these peptides, we evaluated their expression in unlicensed MSC and IFN-*γ*-licensed MSCs exposed to OdDHL. *HAMP* levels were increased in both unlicensed MSC and IFN-*γ*-licensed MSCs cultivated with OdDHL. Under these conditions, IFN-*γ*-licensed MSCs also showed a transcriptional increase in *HBD2*.

To functionally test the antimicrobial potential of unlicensed MSC and IFN-*γ*-licensed MSCs, they were cultured with *P. aeruginosa*. MSCs have antimicrobial properties against both gram-positive and gram-negative bacteria, including *Escherichia coli*, *P. aeruginosa*, and *Staphylococcus aureus*. It has been demonstrated that this activity has been attributed to the production of LL-37 and that this peptide is released at higher levels when MSCs are exposed to bacteria [11]. As expected, unlicensed MSC inhibited the growth of *P. aeruginosa*. However, IFN-*γ*-licensed MSCs showed reduced ability to control bacterial growth. Interestingly, we did not identify any modulation in the expression of genes encoding antimicrobial proteins in MSCs after contact with *P. aeruginosa*. However, in line with what we had observed when cultivating MSCs with OddHL, IFN-*γ*-licensed MSCs were more sensitive and had their viability significantly compromised by culturing with *P. aeruginosa*. Molecularly, IFN-*γ*-licensed MSCs showed high transcriptional levels of *CASP-1* and *BAK* and inhibition of *BCL2*.

## 5. Conclusion

Our *in vitro* findings indicate that the licensing of MSCs with IFN-*γ* can make them more susceptible to cell death when they come into contact with *P. aeruginosa* or quorum-sensing signaling molecules produced by such bacteria. These data are important considering the applicability of MSCs, and based on these findings, it would be important to develop new studies with models of bacterial infections to investigate the anti-inflammatory and antimicrobial potential of licensed MSCs.

## Data Availability

Data will be made available on request.

## Supplementary Materials

Supplementary Materials Table 1: qPCR primer sequences.
