# Supplementary material for: IFN-γ-Licensed Mesenchymal Stem Cells Are More Susceptible to Death when Exposed to Quorum-Sensing Signal Molecule OdDHL and Less Effective in Inhibiting the Growth of Pseudomonas aeruginosa
Source: Stem Cells Int. 2024 Jul 30;2024:2934308. doi: 10.1155/2024/2934308 (PMC11303062; doi:10.1155/2024/2934308)
Supplement: Supplementary Materials — Table 1: qPCR primer sequences. [file 2934308.f1.docx]

**Table1. qPCR primer sequences**

| **Gene** | **Primer** | **Sequence (5’- 3’)** |
| --- | --- | --- |
| *GAPDH* | Foward | TCAACGACCACTTTGTCAAGCTCAGCT |
|  | Reverse | GGTGGTCCAGGGGTCTTAC |
| *TSG-6* | Foward | CCCAGGTTGCTTGGCTGATT |
|  | Reverse | GGACCCATACGTACCTTCCC |
| *IL-10* | Foward | GGCACCCAGTCTGAGAACAG |
|  | Reverse | ACTCTGCTGAAGGCATCTCG |
| *IDO* | Foward | GGGAAGCTTATGACGCCTGT |
|  | Reverse | CTGGCTTGCAGGAATCAGGA |
| *TGF-β* | Forward | GCTGTATTTAAGGACACCGTGC |
|  | Reverse | TGACACAGAGATCCGCAGTC |
| *IFN-γ* | Foward | ACTGTCGCCAGCAGCTAAAA |
|  | Reverse | TATTGCAGGCAGGACAACCA |
| *LCN1* | Foward | GGAGCTGACTTCGGAACTAAAGG |
|  | Reverse | TGTGGTTTTCAGGGAGGCC |
| *HBD2* | Foward | CCAGCCATCAGCCATGAGGG |
|  | Reverse | GGAGCCCTTTCTGAATCCGC |
| *HAMP* | Foward | CCCACAACAGACGGGACAAC |
|  | Reverse | CTCCTTCGCCTCTGGAACAT |
| *LL-37* | Foward | GAAGACCCAAAGGAATGGCC |
|  | Reverse | CAGAGCCCAGAAGCCTGAGC |
| *CASP-1* | Foward | AAGACCCGAGCTTTGATTGACTC |
|  | Reverse | AAATCTCTGCCGACTTTTGTTTCC |
| *CASP-2* | Foward | ACAAAAGTGGCAGTGTGCCT |
|  | Reverse | TCCTTGCTGGTCAACCTGA |
| *BAX* | Foward | CAGACCGTGACCATCTTTGT |
|  | Reverse | GCCTCAGCCCATCTTCTTC |
| *BAK* | Foward | GTTTTCCGCAGCTACGTTTTT |
|  | Reserse | GCAGAGGTAAGGTGACCATCTC |
| *BCL-2* | Foward | CAAAGCTGCAGGCTGTTTAAG |
|  | Reverse | GTCTGTCTGTGTGTGTGATGT |
